# Association Between Optical Coherence Tomography Angiography (OCTA)-Based Retinal Vascular Densities and Empathy in Young Adults

**DOI:** 10.3390/bioengineering12090902

**Published:** 2025-08-22

**Authors:** Bess Yin-Hung Lam, Carole Leung, Ka-Shun Lei, Kaiyip Choi, Henry H. L. Chan

**Affiliations:** 1 Department of Counselling and Psychology, Hong Kong Shue Yan University, Hong Kong SAR, China; 2 Department of Psychology, The University of Texas at Dallas, Richardson, TX 75080-3021, USA; 3 Faculty of Social Sciences, University of Hong Kong, Hong Kong SAR, China; 4 School of Optometry, Hong Kong Polytechnic University, Hong Kong SAR, China; kaiyip.choi@polyu.edu.hk (K.C.); henryhl.chan@polyu.edu.hk (H.H.L.C.)

**Keywords:** empathy, Optical Coherence Tomography Angiography, retinal vessel, perfusion, biomarkers

## Abstract

With the use of Optical Coherence Tomography Angiography (OCTA), the present study is the first study to examine if retinal vascular densities (vessel densities and perfusion densities) could be associated with empathetic levels in young and non-clinical adults. Methods: Fifty-one university students aged from 18 to 25 years (26 males and 24 females) were recruited from a university in Hong Kong. OCTA was conducted to assess their retinal vessel density (VD) and perfusion density (PD) in different scan patterns over the macula (1 mm center subfield, 3 × 3 mm scan, 6 × 6 mm scan). Empathy (cognitive, affective, and somatic) was measured by using the Cognitive, Affective, and Somatic Empathy Scales (CASES). Results: After controlling for age, the multiple linear regression results showed that both the VD and PD in the 1 mm center subfield were significantly and negatively associated with the empathy total score, the affective empathy subscore, and the somatic empathy subscore, respectively (*ps* < 0.05). Conclusion: The present findings indicate that a lower level of empathy is associated with increased retinal vascular densities in the 1 mm center subfield, specifically involving variations in vascular density (VD) and perfusion density (PD). This suggests the dilation of retinal venules might lead to lower empathy. These results establish a foundation for future studies investigating the underlying mechanism of retinal imaging and empathy in healthy individuals.

## 1. Introduction

Empathy is the social cognitive ability to understand and share another person’s feelings [1]. Empathy impairment is also associated with schizophrenia [2]. Previous literature focused on brain imaging studies to examine individuals with these problems [3,4], while the retinal findings were scarce. This study explored whether empathetic problems could be delineated by the approach of retinal imaging, which has a close linkage with brain imaging.

To go beyond current findings from the brain imaging approach, more light has been shed on the investigation of empathy from the retinal imaging methodology in recent years. London, Benhar, and Schwartz [5] described “the retina as a window to the brain.” The retina is an extension of the central nervous system, and its physical structures and functions are similar or closely related to the brain. For instance, brain imaging abnormalities (e.g., white matter lesions, atrophy) and small cerebral artery changes were found to be significantly related to retinal microvascular changes [6]. With regard to the relationship between retinal vessel density (VD), perfusion density (PD), vessel diameter, and retinal nerve fiber layer (RNFL), Geneid et al. [7] found that the narrower the retinal vessels’ diameter (both arteriolar and venular diameters), the thinner the RNFL. These findings suggest that retina VD, PD, vessel diameter, and RNFL are closely related to each other. Moreover, inspired by [8], understanding the vascular mechanisms could further elucidate the physiological underpinnings of empathy, as retinal health is closely linked to cognitive and emotional processes.

In the last decade, besides schizophrenia and other mental disorders [9,10], ophthalmic research has shed more light on the relationship between retinal microvascular changes and cognitive functioning [11,12] but the findings were mixed. For instance, Akin et al. [11] suggested that retinal changes did not predict social cognitive symptoms in patients with schizophrenia, but they might play an important role in identifying high-risk groups. Moreover, wider retinal venules were associated with more psychosis symptoms during childhood and a higher liability to experience psychosis symptoms during adulthood [12]. This might be because insufficient supply of oxygen to the brain can also lead to the dilation of retinal venules [13], suggesting a close relationship between the brain and the retina. Indeed, it would be crucial to investigate empathy in non-clinical individuals instead of focusing on clinical patients through their retinal microvascular characteristics.

With the advancement of retinal imaging techniques such as Optical Coherence Tomography Angiography (OCTA), more ophthalmic studies have been conducted to investigate how the changes in the eyes are associated with psychological functioning and cognitive functioning in recent years [6,9]. OCTA is a suitable method for exploring empathy due to its non-invasive nature, accessibility, and lower participant burden compared to direct brain imaging techniques. It allows for real-time imaging of retinal blood flow, which can correlate with psychological status. Additionally, OCTA provides complementary insights into the peripheral nervous system, enriching the understanding of empathy while facilitating larger and more diverse participant samples. However, little is known about whether retinal microvascular changes are related to empathy, which is closely associated with cognitive function [1]. The present study aimed to examine the relationship between retinal vascular densities, vessel densities (VDs), and perfusion densities (PDs) and empathy in non-clinical young adults, thereby bridging the knowledge between brain and retinal findings. It was hypothesized that VD and PD would be associated with empathy.

## 2. Materials and Methods

### 2.1. Participants

The study was approved by the University Human Subjects Ethics Sub-committee. Fifty-one healthy university students took part in the study, and the mean age of the participants was 21.33 (S.D. = 1.40) (26 males, 51%). One participant did not disclose his or her gender. All 51 healthy participants met a number of inclusion criteria in this study: (1) participants aged between 18 and 25, (2) had good corrected visual acuity, (3) had satisfactory general and ocular health, (4) had no history of retinal vascular pathologies, and (5) were not diagnosed with any psychiatric disorders nor were they undergoing psychiatric medication. Those who reported any history of retinal vascular pathologies were excluded from this study. This selected age group is critical for understanding the development of empathy, allowing researchers to focus on a period of significant emotional and cognitive growth. Having good visual and ocular health helped ensure that findings were more directly related to empathy rather than physical ailments. Excluding individuals with retinal conditions ensured that blood flow measurements were accurate and reflected typical physiological responses rather than pathology, which is key for linking retinal health to empathy. Lastly, screening out those with psychiatric issues ensured that emotional processing was not influenced by mental health issues or medications, allowing for a clearer connection between retinal characteristics and empathy.

### 2.2. Procedures

Written informed consent was obtained from all the participants. Before the administration of the pupil dilation drug, eye assessments of habitual distant visual acuity, intraocular pressure, and the anterior parts (e.g., the cornea) of each eye were conducted. Then, a registered optometrist placed a drop of Mydrin-P (0.5% Tropicamide + 0.5% Phenylephrine) in each eye for dilated fundus examination. The pupil dilation period lasted around 15 to 20 min. Upon the confirmation of the pupil dilation size by the optometrist, 6 mm × 6 mm retinal scans of angiography images were captured from both eyes over the macula via optical coherence tomography angiography (OCTA) (Zeiss CIRRUS HD-OCT 5000, Carl Zeiss Meditec, Inc., Dublin, CA, USA) in a dark room. To be specific, 200 B-scans in a 6 × 6 mm area using macular cube scan mode were acquired in this study. The participants’ retinal blood VD and PD were measured and analyzed according to the Early Treatment Diabetic Retinopathy Study (ETDRS) grid using Zeiss AngioPlex (Carl Zeiss Meditec, Inc., Dublin, CA, USA). The VD and PD measures for analysis were the 1 mm center subfield, 3 × 3 mm scan pattern, and 6 × 6 mm scan pattern. The participants also filled in the Cognitive, Affective, and Somatic Empathy Scales (CASES) [1].

### 2.3. Measures

#### 2.3.1. OCTA: VD and PD

Five indexes (vessel area density (aka perfusion density), vessel skeleton density (aka vessel density), vessel diameter index, vessel perimeter index, and vessel complexity index) can be used to objectively analyze retinal vascular abnormalities from OCT angiogram (OCTA) scans [14]. VD is defined as the ratio of the total length of the blood vessels to the total image area of a region of measurement [14]. PD represents the ratio of the total area occupied by blood vessels to the total image area in a region of measurement [14].

**Figure 1:**
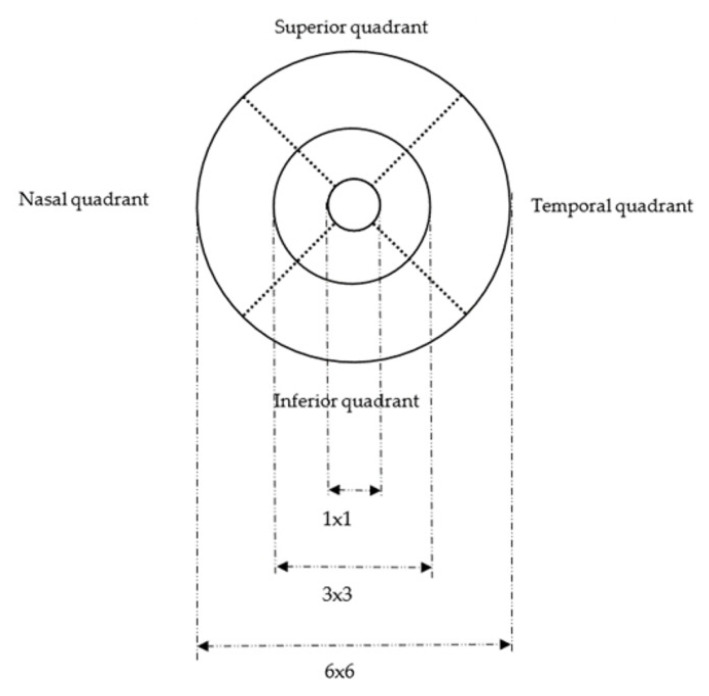
A diagram of the OCTA scan patterns. Note. This is a diagram showing the Early Treatment Diabetic Retinopathy Study (ETDRS) grid region overlay used for calculating the retinal vessel density (VD) and perfusion density (PD) in the 1 × 1 mm center subfield, 3 × 3 mm scan pattern, and 6 × 6 mm scan pattern.

Three commonly used OCTA scan patterns were acquired using an ETDRS grid, which includes the 1 mm center subfield, 3 × 3 mm scan pattern, and 6 × 6 mm scan pattern [15,16]. For the data analysis, we combined the vascular density measurements obtained from both the left and right eyes (by summation) for each participant (51 participants) across all scan patterns. The densities of the four quadrants (superior, nasal, temporal, and inferior quadrant) were also computed for each of the following scan patterns: 3 × 3 mm scan, 6 × 6 mm scan (Figure 1). We adopted several previous research in analyzing all four quadrant estimates [15,16,17].

#### 2.3.2. Cognitive, Affective, and Somatic Empathy Scales (CASES)

Empathy was measured via CASES [1] with a three-factor structure (cognitive empathy, affective empathy, and somatic empathy). Cognitive empathy refers to the ability to understand others’ feelings. Affective empathy refers to the ability to perceive others’ feelings. Somatic empathy refers to the automatic bodily response to an observed event. A lower score on the scales suggests that the participant has a deficit in empathic abilities.

#### 2.3.3. Statistical Analysis

A summation of the VD and PD measures from both eyes and the four quadrants were computed as mentioned above and 51 pairs of eyes were included for the data analysis. In order to test whether retinal vascular densities were associated with the level of empathy among young adults, multiple linear regression analyses were conducted. The dependent variable for all the regression analyses was empathy. The regression analyses were conducted separately for each retinal measure paired with each CASES score.

## 3. Results

### 3.1. Descriptive Statistics

The mean age was 21.33 years (S.D. = 1.40 years). The means of empathy (CASES) total, cognitive, affective, and somatic scores were 36.56, 12.76, 12.84, and 10.82, respectively (Table 1). With regard to the retinal vascular densities (VDs), the means of the 1 mm center subfield, 3 × 3 mm scan pattern, and 6 × 6 mm scan pattern were 20.41 (S.D. = 4.94), 146.62 (S.D. = 8.12), and 150.76 (S.D. = 5.35). Regarding the PD estimates, the means of the 1 mm center subfield, 3 × 3 mm scan pattern, and 6 × 6 mm scan pattern were 0.45 (S.D. = 0.11), 3.46 (S.D. = 0.20), and 3.70 (S.D. = 0.12).

### 3.2. Correlations and Regression Analyses

Significant negative correlations were found between the VD and PD in the 1 mm center subfield and CASES-total score, affective score, and somatic score (range of *r* = −0.34 to −0.30, *ps* < 0.05) (Table 2).

After controlling for age, the CASES-total score was significantly and negatively associated with VD in the 1 mm center subfield (*R*^2^ = 0.16, *F*(2,47) = 4.46, *p* = 0.02, *β* = −0.28, *t*(47) = −2.12, *p* = 0.04) and PD in the same region (*R*^2^ = 0.17, *F*(2,47) = 4.65, *p* = 0.01, *β* = −0.29, *t*(47) = −2.20, *p* = 0.03) (Table 3 and Table 4). The results for the relationship between VD and PD in 3 × 3 mm and 6 × 6 mm scan patterns were not significant (*ps* > 0.05) (Table 3 and Table 4). In terms of the three empathy subscores, significant results were found for the VD and PD measures in the 1 mm center subfield in relation to CASES-affective and CASES-somatic empathy (*ps* < 0.05). Specifically, the VD and PD in the 1 mm center subfield were significant predictors of the CASES-affective score (VD: *R*^2^ = 0.13, *F*(2,47) = 3.42, *p* = 0.04, *β* = −0.30, *t*(47) = −2.19, *p* = 0.03; PD: *R*^2^ = 0.13, *F*(2,47) = 3.42, *p* = 0.04, *β* = −0.30, *t*(47) = −2.19, *p* = 0.03). The CASES-somatic score was significantly and negatively associated with VD in the 1 mm center subfield (*R*^2^ = 0.14, *F*(2,48) = 4.03, *p* = 0.02, *β* = −0.32, *t*(48) = −2.35, *p* = 0.02) and PD in the same region (*R*^2^ = 0.15, *F*(2,48) = 4.30, *p* = 0.02, *β* = −0.33, *t*(48) = −2.46, *p* = 0.02). With the significance threshold after the Bonferroni correction (*p* < 0.025), all these results became marginally significant.

## 4. Discussion

To the authors’ best knowledge, the current study was the first to examine the relationship between retinal vascular densities (vessel densities and perfusion densities) and empathetic levels in healthy young adults. The VD and PD in the 1 mm center subfield over the macula were negatively associated with the affective empathy and somatic empathy levels, suggesting that the dilation of retinal venules might lead to lower empathy. The present findings not only set the scientific foundation for investigating the relationship between retinal characteristics and empathy in the field; they also suggest that retinal microvascular abnormality can potentially be used as a biomarker for the detection of those with a lack of empathy.

A significant relationship between retinal vasculature and empathy found in the present study supports the notion that retinal microvasculature is closely related to empathy. Specifically, the current results show that both the VD and PD in the 1 mm center subfield were significantly and negatively associated with the empathy total score, the affective empathy subscore, and the somatic empathy subscore, respectively. Indeed, previous findings suggested that wider retinal venules were found to be associated with more psychosis symptoms during childhood, with a higher likelihood to experience psychosis symptoms during adulthood [12]. This might be because an insufficient supply of oxygen to the brain can also lead to the dilation of retinal venules [18], suggesting a close relationship between the brain and the retina and also the potential inverse relationship between wider vessels and empathy. Furthermore, conditions associated with increased retinal vascularization, such as inflammation [19], may negatively affect brain areas responsible for empathy, leading to a decrease in empathetic responses. Moreover, Al-Mazidi [20] suggested that Disintegrin and metalloproteinase domain-containing protein 10 (ADAM10) and ciliary neurotrophic factor (CNTF), which could facilitate the shedding of membrane proteins involved in vascular signaling, affecting the development and maintenance of retinal vessels, were biomarkers of schizophrenia and autism spectrum disorder who often had lower levels of empathy. The current findings on the relationship between retinal vascular densities and empathy was based on a non-clinical sample. This result diverges from previous ophthalmic research [9] conducted on schizophrenia patients and individuals with high trait anger, who often exhibit impaired empathetic skills, highlighting the broader implications of retinal health in understanding empathy beyond clinical populations.

More importantly, the present findings extend the previous literature on the relationship between cognitive function and retinal characteristics by establishing the relationship between empathy (affective and somatic empathy) and specific retinal microvascular characteristics (the VD and PD in the 1 mm center subfield). Specifically, previous research has focused on cognitive functions and found that retinal vascular abnormalities were associated with cognitive impairment (e.g., memory, processing speed, executive functioning) and psychological indexes (e.g., insomnia, activities of daily living, and deterioration) [6,21,22]. For instance, Girbardt et al. [21] reported that thinner retinal nerve fiber layer thickness was found to be a meaningful index for poorer cognitive performance, which presents the potential for the prediction of future cognitive decline, which was in turn related to a lower level of empathy [23].

There are several possible explanations underlying the relationship between PD and VD and empathy (affective and somatic empathy) found in the current sample. Specifically, an insufficient supply of oxygen to the brain and retina tissues or structural damage due to inflammation or endothelial dysfunction may negatively impact social cognitive functioning and emotional responses [12,18]. For instance, the retinal vascular changes detected by OCTA could be a reflection of social cognitive changes resulting due to structural alterations in the brain, such as in the prefrontal cortex and amygdala, which are integral to empathy. Along the same line, inflammatory processes can lead to changes in vascular permeability and the integrity of the blood–brain barrier [24], which can hinder the delivery of essential nutrients and oxygen to neural tissues. This dysfunction can contribute to neurodegeneration [25] and affect the neural networks responsible for social cognitive functioning. All these might explain the significant association between retinal characteristics and empathy levels. However, it is important to note that the retinal characteristics were not found to be significantly associated with cognitive empathy. This may be due to the fact that cognitive empathy is more influenced by age, rather than by retinal vasculatures, which was supported by our current data, which is consistent with Del Rey et al.’s [26] findings.

### Limitations

There are a number of limitations that should be addressed in future studies. For instance, the underlying mechanism of the relationship between retinal vasculature and empathy is yet to be investigated. Future studies should also administer neuroimaging methods to investigate brain structural change and cerebral blood flow information such as Magnetic Resonance Imaging (MRI) scans in order to help gain a better understanding of how the retina acts as a window to the brain in relation to empathy. This integration would provide valuable insights into how retinal characteristics reflect cerebral health and functionality, enhancing our understanding of the retina as a “window to the brain” concerning empathy. Second, the current study has a relatively small sample size with a cross-sectional design. The relationship between retinal characteristics and empathy has never been studied before and current findings can provide important information for larger-scale studies in this area in the future. Hence, future studies might investigate the temporal relationship between retinal vasculature and empathy with a larger sample size. Last but not least, the current study only investigated 18–25-year-old individuals because we would like to focus on early young adulthood in which the brain and visual system are reaching its maturity level. Since the age range was limited, current findings might be mostly applicable to 18–25-year-old individuals and future studies might investigate a wider age range for better generalization of the results.

## 5. Conclusions

These key findings indicate that retinal microvascular abnormality can potentially be used as a biomarker for the detection of those with a lack of empathy including schizophrenia. Identifying retinal microvascular changes as potential biomarkers for empathy can facilitate early detection of social cognitive deficits. This is particularly important since empathy is crucial for interpersonal relationships and overall mental health. Early intervention strategies could be developed to address these deficits before they lead to more severe psychological issues. Moreover, the association between retinal health and empathy underscores the importance of maintaining vascular health for overall psychological well-being. This could prompt public health initiatives focused on education about the relationship between physical health and psychological well-being. Although more precise segmentation of different layers in the fundus is provided by OCTA when compared with FA, it is noteworthy that the combinatorial approaches of OCTA and FA could be more accurate in detecting those with a lack of empathy in a clinical setting.

## Figures and Tables

**Table 1:** Descriptive statistics of all the variables in the present study.

Variables		Mean	SD
CASES (empathy scores)	Total score (Range= 9–58)	36.56	9.95
	Cognitive score	12.76	3.70
	Affective score	12.84	3.79
	Somatic score	10.82	3.78
Retinal vascular densities	VD in the 1 mm center subfield	20.41	4.94
	VD in the 3 × 3 mm scan pattern	146.62	8.12
	VD in the 6 × 6 mm scan pattern	150.76	5.35
	PD in the 1 mm center subfield	0.45	0.11
	PD in the 3 × 3 mm scan pattern	3.46	0.20
	PD in the 6 × 6 mm scan pattern	3.70	0.12
Age		21.33	1.40

Note. VD, vessel density; PD, perfusion density; CASES, Cognitive, Affective, and Somatic Empathy Scales; SD, standard deviation.

**Table 2:** Correlations between the variables in the present study.

Major Variables	1	2	3	4	5	6	7	8	9	10	11
1. Age	1	-	-	-	-	-	-	-	-	-	-
2. VD in the 1 mm center subfield	−0.06	1	-	-	-	-	-	-	-	-	-
3. VD in the 3 × 3 mm scan pattern	−0.03	0.51 ***	1	-	-	-	-	-	-	-	-
4. VD in the 6 × 6 mm scan pattern	0.15	0.25	0.67 ***	1	-	-	-	-	-	-	-
5. PD in the 1 mm center subfield	−0.05	0.995 ***	0.54 ***	0.27	1	-	-	-	-	-	-
6. PD in the 3 × 3 mm scan pattern	−0.10	0.48 ***	0.98 ***	0.64 ***	0.51 ***	1	-	-	-	-	-
7. PD in the 6 × 6 mm scan pattern	0.09	0.15	0.69 ***	0.72 ***	0.20	0.71 ***	1	-	-	-	-
8. CASES-Total	0.28 *	−0.30 *	−0.15	−0.14	−0.30 *	−0.12	−0.07	1	-	-	-
9. CASES-Cognitive	0.28 *	−0.13	−0.03	−0.02	−0.14	−0.01	0.01	0.83 ***	1	-	-
10. CASES-Affective	0.20	−0.31 *	−0.16	−0.19	−0.31 *	−0.14	−0.09	0.91 ***	0.61 ***	1	-
11. CASES-Somatic	0.21	−0.33 *	−0.17	−0.16	−0.34 *	−0.15	−0.09	0.90 ***	0.60 ***	0.79 ***	1

Note. * *p* < 0.05, *** *p* < 0.001.; VD, vessel density; PD, perfusion density; CASES, Cognitive, Affective, and Somatic Empathy Scales.

**Table 3:** Multiple linear regression analyses for the relationship between empathy (measured by CASES) and retinal vessel density (VD).

Model	D.V. (Empathy)	I.V. (VD)	*R* ^2^	*F (df)*	*p*	*β*	*t*	*p*	Controlling for Age	*R* ^2^	*F (df)*	*p*	*β*	*t*	*p*
1	CASES-total	1 mm center subfield	0.088	4.64 (1,48)	0.04 *	−0.30	−2.15	0.04 * ^		0.160	4.46 (2,47)	0.02	−0.28	−2.12	0.04 *
		Age											0.27	2.00	0.05
2	CASES-total	3 × 3 mm scan	0.021	1.04 (1,48)	0.31	−0.15	−1.02	0.31		0.099	2.59 (2,47)	0.09	−0.14	−1.02	0.311
		Age											0.28	2.02	0.05 *
3	CASES-total	6 × 6 mm scan	0.021	1.01 (1,48)	0.32	−0.14	−1.01	0.32		0.115	3.07 (2,47)	0.06	−0.19	−1.39	0.17
		Age											0.31	2.24	0.03 *
4	CASES-cognitive	1 mm center subfield	0.017	0.84 (1,49)	0.36	−0.13	−0.92	0.36		0.093	2.47 (2,48)	0.10	−0.11	−0.83	0.41
		Age											0.28	2.01	0.05
5	CASES-cognitive	3 × 3 mm scan	0.001	0.06 (1,49)	0.82	−0.03	−0.024	0.82		0.081	2.11 (2,48)	0.13	−0.03	−0.18	0.86
		Age											0.28	2.04	0.05 *
6	CASES-cognitive	6 × 6 mm scan	0.000	0.02	0.89	−0.02	−0.14	0.89		0.084	2.20 (2,48)	0.12	−0.06	−0.45	0.66
		Age											0.29	2.09	0.04 *
7	CASES-affective	1 mm center subfield	0.094	5.0 (1,48)	0.03 *	−0.31	−2.24	0.03 * ^		0.127	3.42 (2,47)	0.04 *	−0.30	−2.19	0.03 *
		Age											0.18	1.33	0.19
8	CASES-affective	3 × 3 mm scan	0.02	1.20 (1,48)	0.28	−0.16	−1.09	0.28		0.062	1.54 (2,47)	0.23	−0.15	−1.08	0.28
		Age											0.19	1.37	0.18
9	CASES-affective	6 × 6 mm scan	0.03	1.69 (1,48)	0.20	−0.19	−1.30	0.20		0.086	2.20 (2,47)	0.12	−0.22	−1.56	0.125
		Age											0.23	1.63	0.11
10	CASES-somatic	1 mm center subfield	0.11	5.82 (1,49)	0.02 *	−0.33	−2.41	0.02 * ^		0.144	4.03 (2,48)	0.02 *	−0.32	−2.35	0.02 *
		Age											0.19	1.45	0.15
11	CASES-somatic	3 × 3 mm scan	0.03	1.45 (1,49)	0.23	−0.17	−1.21	0.23		0.072	1.86 (2,48)	0.17	−0.16	−1.18	0.25
		Age											0.21	1.49	0.14
12	CASES-somatic	6 × 6 mm scan	0.03	1.25 (1,49)	0.27	−0.16	−1.12	0.27		0.081	2.12 (2,48)	0.13	−0.19	−1.38	0.18
		Age											0.24	1.72	0.09

Note. *p < 0.05, ^ marginally significant with Bonferroni correction. VD, vessel density; CASES, Cognitive, Affective, and Somatic Empathy Scales.

**Table 4:** Multiple linear regression analyses for the relationship between empathy (measured by CASES) and retinal perfusion density (PD).

Model	D.V. (Empathy)	I.V. (PD)	*R* ^2^	*F (df)*	*p*	*β*	*t*	*p*	Controlling for Age	*R* ^2^	*F (df)*	*p*	*β*	*t*	*p*
1	CASES-total	1 mm center subfield	0.092	4.88 (1,48)	0.03 *	−0.30	−2.21	0.03 * ^		0.165	4.65 (2,47)	0.01 *	−0.29	−2.20	0.03 *
		Age											0.27	2.03	0.05 *
2	CASES-total	3 × 3 mm scan	0.015	0.74 (1,48)	0.39	−0.12	−0.86	0.39		0.089	2.31 (2,47)	0.11	−0.10	−0.73	0.47
		Age											0.27	1.96	0.06
3	CASES-total	6 × 6 mm scan	0.005	0.24 (1,47)	0.63	−0.07	−0.48	0.63		0.085	2.13 (2,46)	0.13	−0.10	−0.71	0.49
		Age											0.28	2.01	0.05
4	CASES-cognitive	1 mm center subfield	0.019	0.93 (1,49)	0.34	−0.014	−0.96	0.34		0.095	2.53 (2,48)	0.09	−0.12	−0.090	0.38
		Age											0.28	2.02	0.05 *
5	CASES-cognitive	3 × 3 mm scan	0.000	0.004 (1,49)	0.95	−0.01	−0.06	0.95		0.080	2.10 (2,48)	0.13	0.02	0.14	0.89
		Age											0.29	2.05	0.05 *
6	CASES-cognitive	6 × 6 mm scan	0.000	0.001 (1,48)	0.97	0.01	0.04	0.97		0.073	1.84 (2,47)	0.17	−0.02	−0.14	0.89
		Age											0.27	1.92	0.06
7	CASES-affective	1 mm center subfield	0.093	4.92 (1,48)	0.03 *	−0.31	−2.22	0.03 * ^		0.127	3.42 (2,47)	0.04 *	−0.30	−2.19	0.03 *
		Age											0.18	1.35	0.18
8	CASES-affective	3 × 3 mm scan	0.019	0.93 (1,48)	0.34	−0.14	−0.97	0.34		0.053	1.32 (2,47)	0.28	−0.12	−0.87	0.39
		Age											0.19	1.30	0.20
9	CASES-affective	6 × 6 mm scan	0.007	0.35 (1,47)	0.56	−0.09	−0.59	0.56		0.049	1.17 (2,46)	0.32	−0.11	−0.74	0.46
		Age											0.20	1.41	0.17
10	CASES-somatic	1 mm center subfield	0.113	6.27 (1,49)	0.02 *	−0.34	−2.50	0.02 * ^		0.152	4.30 (2,48)	0.02 *	−0.33	−2.46	0.02 *
		Age											0.20	1.48	0.15
11	CASES-somatic	3 × 3 mm scan	0.022	1.10 (1,49)	0.30	−0.15	−1.05	0.30		0.062	1.57 (2,48)	0.22	−0.13	−0.92	0.36
		Age											0.20	1.42	0.16
12	CASES-somatic	6 × 6 mm scan	0.008	0.40 (1,48)	0.53	−0.09	−0.63	0.53		0.056	1.40 (2,47)	0.26	−0.11	−0.78	0.44
		Age											0.22	1.55	0.13

Note. *p < 0.05, ^ marginally significant with Bonferroni correction. PD, perfusion density; CASES, Cognitive, Affective, and Somatic Empathy Scales.

## Data Availability

Data is available upon request.

## Abbreviations

The following abbreviations are used in this manuscript:

OCTA	Optical Coherence Tomography Angiography
VD	Vessel Density
PD	Perfusion Density
CASES	Cognitive, Affective, and Somatic Empathy Scales
